# Insights into prokaryotic communities and their potential functions in biogeochemical cycles in cold seep

**DOI:** 10.1128/msphere.00549-24

**Published:** 2024-09-13

**Authors:** Qiumei Quan, Jiaxing Liu, Chaolun Li, Zhixin Ke, Yehui Tan

**Affiliations:** 1 South China Sea Institute of Oceanology, Chinese Academy of Sciences, Guangzhou, China; 2 University of Chinese Academy of Sciences, Beijing, China; University of Wisconsin-Madison, Madison, Wisconsin, USA

**Keywords:** cold seep, prokaryotes, biogeochemical cycles, dissolved organic matter

## Abstract

Microorganisms are significant drivers of organic matter mineralization and are essential in marine biogeochemical cycles. However, the variations and influencing factors in prokaryotic communities from cold-seep sediments to the water column and the specific role of these microorganisms in biogeochemical cycles in the water column above cold seep remain unclear. Here, we investigated prokaryotic communities and their roles in nitrogen/sulfur cycling processes and conducted *in situ* dissolved organic matter (DOM) enrichment experiments to explore the effects of diverse sources of DOM on prokaryotic communities. Field investigations showed that the prokaryotic communities in the near-bottom water were more similar to those in the deep layer of the euphotic zone (44.60%) and at a depth of 400 m (50.89%) than those in the sediment (18.00%). DOM enrichment experiments revealed that adding dissolved organic nitrogen (DON) and phosphorus DOP caused a notable increase in the relative abundances of Rhodobacterales and Vibrionales, respectively. A remarkable increase was observed in the relative abundance of Alteromonadales and Pseudomonadales after the addition of dissolved organic sulfur (DOS). The metagenomic results revealed that Proteobacteria served as the keystone taxa in mediating the biogeochemical cycles of nitrogen, phosphorus, and sulfur in the Haima cold seep. This study highlights the responses of prokaryotes to DOM with different components and the microbially driven elemental cycles in cold seeps, providing a foundational reference for further studies on material energy metabolism and the coupled cycling of essential elements mediated by deep-sea microorganisms.

**IMPORTANCE:**

Deep-sea cold seeps are among the most productive ecosystems, sustaining unique fauna and microbial communities through the release of methane and other hydrocarbons. Our study revealed that the influence of seepage fluid on the prokaryotic community in the water column is surprisingly limited, which challenges conventional views regarding the impact of seepage fluids. In addition, we identified that different DOM compositions play a crucial role in shaping the prokaryotic community composition, providing new insights into the factors driving microbial diversity in cold seeps. Furthermore, the study highlighted Proteobacteria as key and multifaceted drivers of biogeochemical cycles in cold seeps, emphasizing their significant contribution to complex interactions and processes. These findings offer a fresh perspective on the dynamics of cold-seep environments and their microbial communities, advancing our understanding of the biogeochemical functions in deep-sea environments.

## INTRODUCTION

Deep-sea cold seeps are productive ecosystems dominated by chemoautotrophic microorganisms, in which deeply sourced hydrocarbon- and hydrogen-sulfide-rich fluids are discharged at the seafloor (1). These compounds can reach approximately 100 m above the seafloor and alter the environmental characteristics of the water column, such as pH reduction and decreased dissolved oxygen (DO) (2–4), affecting the microbial community distribution and biogeochemical cycles (5, 6). Recent studies of cold-seep microbiology have expanded beyond sulfate-reducing bacteria and anaerobic methane-oxidizing archaea in sediments (7, 8). Given the significance of microorganisms in cold-seep water columns in mitigating methane emissions and facilitating biogeochemical cycles (4), there has been a growing focus on the microbial community structure in water columns above cold seeps (9–11). A previous study indicated that in the Haima and F cold seeps of the South China Sea (SCS), in addition to chemolithoautotrophic organisms, heterotrophic prokaryotes (primarily Alphaproteobacteria and Gammaproteobacteria) are significant taxa in deeper water layers (10). Seepage intensity, pH, temperature, and nutrients markedly influence the prokaryotic communities and assemblage in the cold-seep water column (6, 9). In addition, the settling of organic particles formed in the euphotic zone can enhance the vertical connectivity of marine microbial communities, influencing the composition of deep-sea microbial communities (12). Consequently, prokaryotic communities in cold-seep water columns may be subject to dual influences from the settling of upper-layer particulate matter and seepage activity. However, it is unclear whether the prokaryotic communities in cold-seep bottom water are more similar to those in the upper water or those in sediments.

Microorganisms rely predominantly on organic matter as carbon and energy sources (13). In cold-seep environments, organic matter originates from the sinking of photosynthetic products from the upper ocean (14, 15) and the release of chemosynthetic products within the sediment (16, 17). Settling organic matter may undergo processes such as escape, disaggregation, dissolution, and solubilization, resulting in the formation of diverse dissolved organic matter (DOM) components (18). Different types of organic matter may attract or inhibit specific prokaryotic microorganisms, resulting in alterations to community structure (19). For example, high-molecular-weight compounds, such as algal polysaccharides, proteins, and glycoproteins, promote the growth of Alteromonadaceae (20, 21), whereas the Roseobacter clade predominates in the utilization of low-molecular-weight organic compounds such as polyamines and taurine (22, 23). In turn, these prokaryotes acquire nutrients through the decomposition and mineralization of organic matter, thereby influencing the biogeochemical cycles of elements and ecosystem stability (24, 25). However, the response of prokaryotic communities to DOM and how these microorganisms mediate and drive the nitrogen, phosphorus, and sulfur cycles in cold-seep water columns are partially understood.

Environmental variations can affect the microbial community structure, thereby altering biogeochemical element cycling processes. In cold seeps, seepage influences the environmental conditions of the water column (2–4, 6, 9). However, the prokaryotic community structure in water columns with varying seepage intensities remains unclear. We hypothesized that there are significant differences in the composition of prokaryotic communities and their associated biogeochemical cycle processes between active and non-active zones, and prokaryotes in the sediment may be released into the water column through seepage fluid. In this study, we analyzed the composition of prokaryotic communities and their contribution to nitrogen, phosphorus, and sulfur cycle processes in the Haima cold seep using high-throughput amplicon sequencing (16S rRNA) and metagenomic techniques. In addition, given the significance of organic matter as a primary energy source for deep-sea microorganisms, it may directly influence the composition and ecological functions of microbial communities. Therefore, we hypothesized that organic matter will alter the prokaryotic community composition in cold-seep waters column and that different organic matter components will have varying effects. To test this hypothesis, we conducted *in situ* enrichment experiments with different DOM compositions at depths of 150–200 m in the euphotic zone and 1,300–1,400 m near the seabed to elucidate the response of prokaryotic communities in cold seeps to organic matter inputs.

## RESULTS

### Environmental conditions

**Fig 1:**
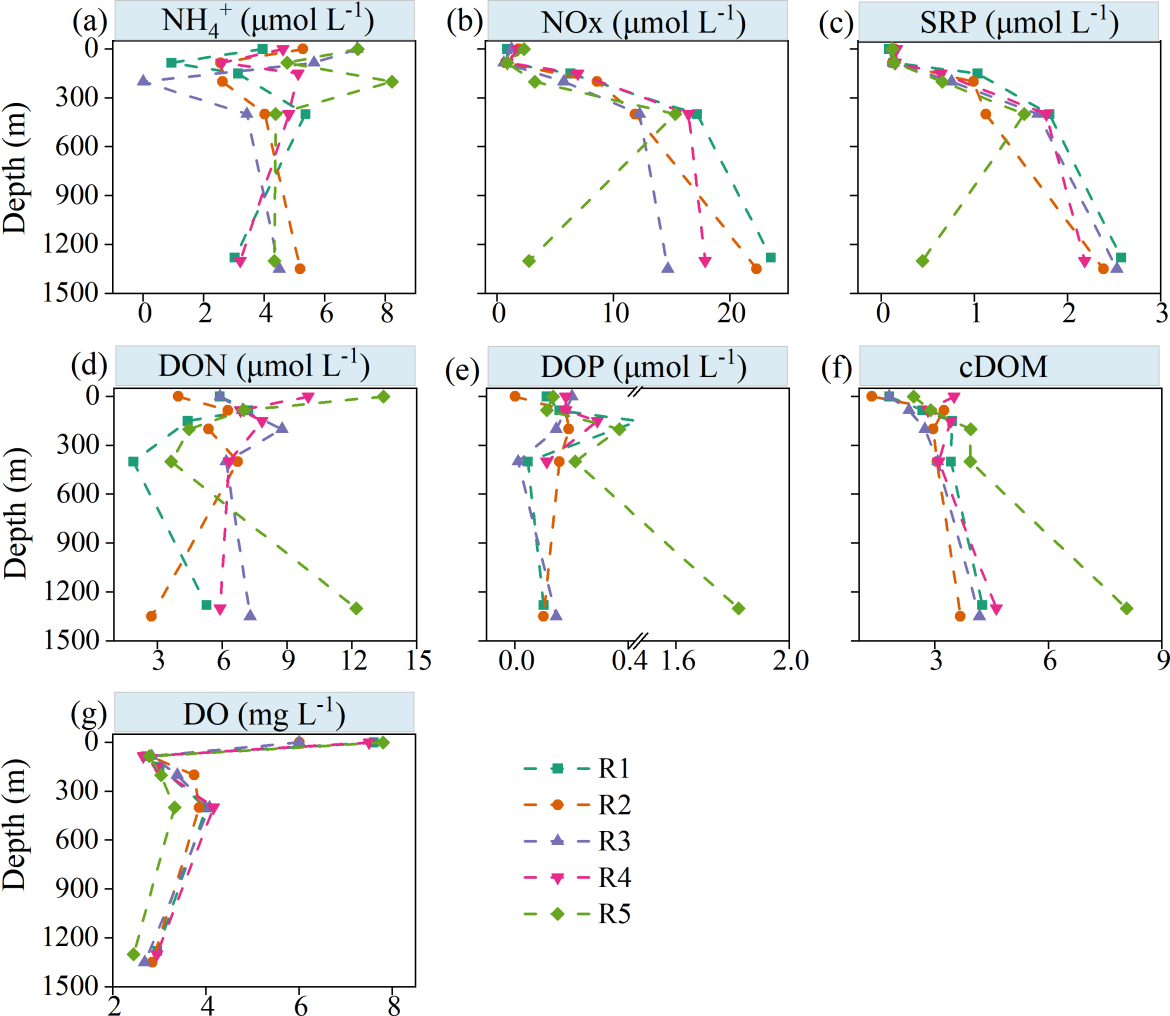
Vertical profile of environmental parameters at five sites in the water column above the Haima cold seep: NH_4_^+^, NO_x_ (NO_2_^−^ + NO_3_^−^), SRP, DOP, DON, cDOM, and DO. SRP: soluble reactive phosphate; DON, dissolved organic nitrogen; DOP, dissolved organic phosphorus; cDOM, chromophoric dissolved organic matter; DO, dissolved oxygen.

The concentrations of inorganic nutrients (NH_4_^+^, NO_x_, and SRP) in R1–R4, except for R5, exhibited an increasing trend from the surface to the bottom layer of the water column (Fig. 1). However, the concentrations of DOP and DON, as well as the relative content of cDOM, were higher at the bottom, especially in R5, with concentrations of 1.82 µmol L^−1^ (DOP), 12.19 µmol L^−1^ (DON), and 8.07 (cDOM), respectively. DO shows a pattern of initially decreasing with depth, then increasing, and finally decreasing again (Fig. 1). Low peaks were observed at 85 m depth in the euphotic zone and near the sediment at the bottom, with average concentrations of 2.75 µmol L^−1^ and 2.77 µmol L^−1^, respectively (Fig. 1).

### Vertical variation of prokaryotic community composition and diversity

**Fig 2:**
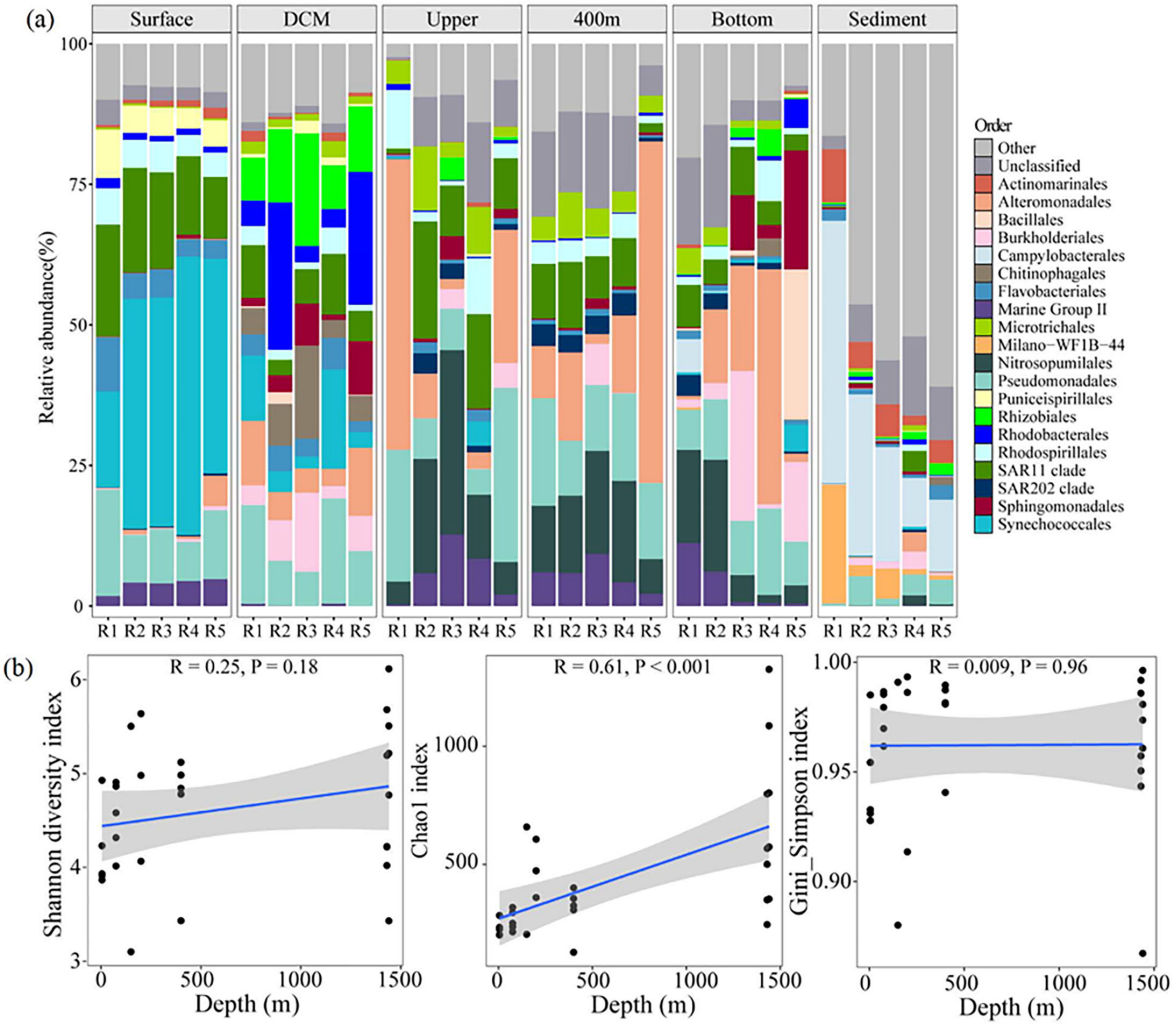
Prokaryotic community structure based on 16s rRNA in the Haima cold seep. (**a**) The relative abundance of dominant taxa (top 20) at the order level at each sample. (**b**) Linear correlation between alpha diversity indices (Shannon, Chao1, and Gini_Simpson) of the whole prokaryotic community and depth.

The 16S rRNA gene analysis showed that the dominant taxa in the surface layers were Synechococcales (17.04%–49.47%), SAR11 clade (11%–19.83%), and Pseudomonadales (6.94%–18.90%; Fig. 2a). Rhizobiales and Rhodobacterales dominated the DCM depth, with Rhodobacterales exhibiting substantially higher relative abundances at R2 (26.19%) and R5 (23.59%) than the average across other sites (3.57%). The prokaryotic communities in the upper, 400 m, and bottom layers shared similar compositions, primarily comprising the SAR11 clade, Pseudomonadales, Alteromonadales, Microtrichales, Nitrosopumilales archaea, and marine group II. Discernible differences were observed in the predominant taxa between the active and non-active zones of the bottom layers (average dissimilarity: 66.49%). The active zones were characterized by a higher relative abundance of archaea Nitrosopumilales (16.59%–19.90%), whereas the non-active zones, represented by R3, R4, and R5, were dominated by Burkholderiales (26.67%), Alteromonadales (41.79%), Bacillales (26.69%), and Sphingomonadales (21.13%). The primary taxa observed in sediment across all sites were Campylobacterales, Actinomarinales, and Milano-WF1B-44 (Fig. 2a). The Chao1 index (*P* < 0.001) significantly increased with depth, but the Shannon diversity (*P* = 0.18) and Gini_Simpson (*P* = 0.96) indices did not show a significant trend (Fig. 2b).

**Fig 3:**
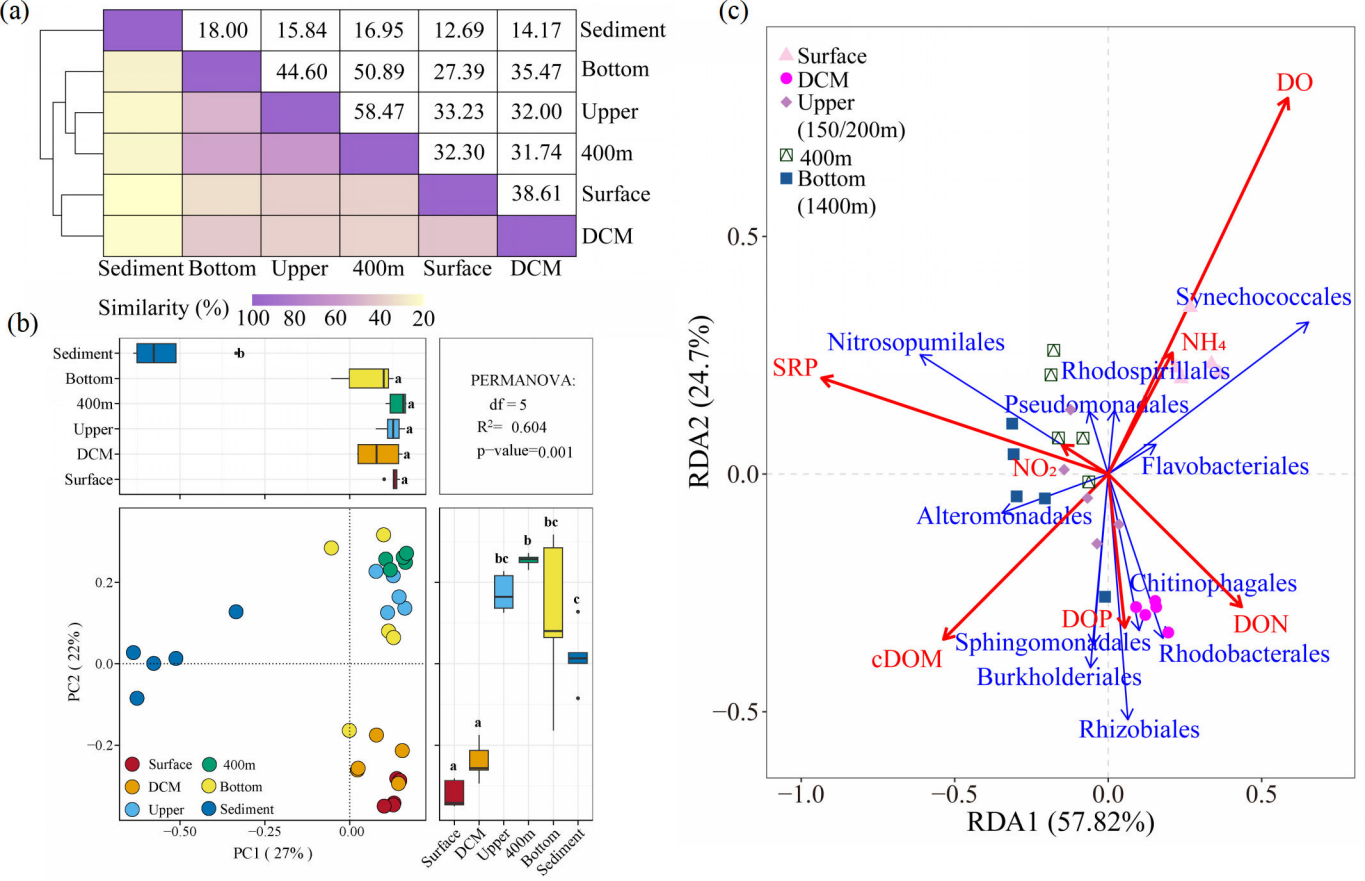
(**a**) SIMPER analysis (Similarity measure: Bray–Curtis) shows the similarity of taxa in the relative abundance between each depth based on the order level. (**b**) PCoA of beta diversity (Bray–Curtis distances) at the order level. Permutational multivariate analysis of variance was used to estimate the significance of differences among the samples. Different letters indicate significant differences (*P* < 0.05). (**c**) Redundancy analysis between main taxa at the order levels and environmental factors (log-transformed) in the water column above the Haima cold seep. Multicollinearity was tested using the variance inflation factor (VIF), and the variables with a VIF >10 were removed.

SIMPER analysis revealed that the similarity of prokaryotic communities among these three layers was the highest, ranging from 44.60% (upper and bottom) to 58.47% (upper and 400 m; Fig. 3a). Principal coordinate analysis (PCoA) further showed the spatial distribution patterns of microbial communities across different sampling depths. The results indicated that samples from the same depth tended to cluster together. The prokaryotic communities in the upper, 400 m, and bottom layers were similar (Fig. 3b). Redundancy analysis (RDA) was performed to identify possible relationship between prokaryotic community composition and environmental variables (Fig. 3c). The results showed that SRP (*R*^2^ = 0.714, *P* < 0.01), DO (*R*^2^ = 0.703, *P* < 0.01), and cDOM (*R*^2^ = 0.314, *P* < 0.05) were the most important factors affecting the prokaryotic community composition. Specifically, the order Alteromonadales was positively correlated with cDOM, whereas Rhodobacterales and Rhizobiales were positively correlated with DON and DOP. Synechococcales exhibited a positive correlation with DO and NH_4_^+^ concentrations (Fig. 3c).

### Response of the prokaryotic community structure to DOM (containing nitrogen, phosphorus, sulfur, and carbon) addition

**Fig 4:**
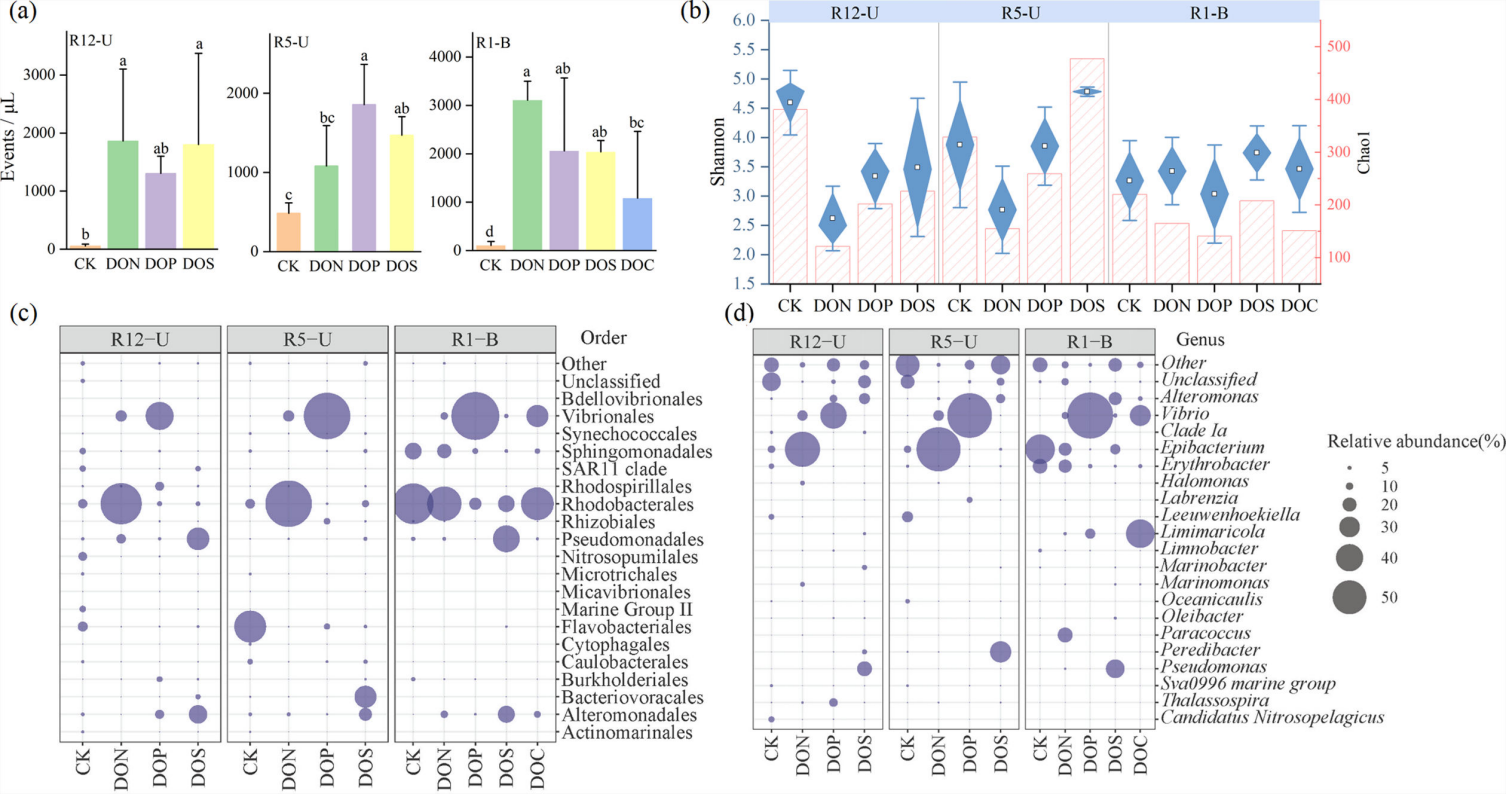
Response of prokaryotic community structure to the addition of different DOM compositions. (**a**) Change in the heterotrophic bacteria abundance and (**b**) the alpha-diversity indices. (**c and d**) The percentage of relative abundances for the top 20 abundant microorganisms at the order and genus levels. CK, no DOM addition; DOC, dissolved organic carbon; DON, dissolved organic nitrogen; DOP, dissolved organic phosphorus; DOS, dissolved organic sulfur.

We further verified the response of prokaryotic communities to DOM with different components using *in situ* enrichment experiments. There was a significant increase in the abundance of heterotrophic bacteria after DOM addition (*P* < 0.05; Fig. 4a). However, the diversity indices (Shannon and Chao1) of the prokaryotic community decreased, except in the DOS treatment in R5-U (Fig. 4b). Rhodobacterales dominated the relative abundance after adding DON. DOP consistently increased the relative abundance of Vibrionales (39.65%–68.53%). In addition, Pseudomonadales and Alteromonadales were more abundant after DOS addition (Fig. 4c). However, Flavobacteriales (in R12-U and R5-U), as well as Sphingomonadales and Rhodobacterales (in R1-B), exhibited decreasing trends in their relative abundance in the DOM treatments (Fig. 4c).

Co-occurrence networks of prokaryotic communities for four treatments (CK, DON, DOP, and DOS) were constructed (Fig. S1a and b). The results indicated that the prokaryotic composition of the top six modules (with a high degree) changed after the addition of DOM. In the CK treatment, the communities primarily comprised Flavobacteriales and SAR11 clade, whereas, in the DON treatment, it was dominated by Vibrionales, Rhodobacterales, and Alteromonadales. The DOP treatment primarily comprised Vibrionales and Burkholderiales, while the DOS treatment was predominantly represented by Alteromonadales and Pseudomonadales. The topological properties of the DOM treatment were lower than those of the CK treatment (nodes: 294, edges: 4947; Fig. S1c), with the DON treatment exhibiting the lowest values (nodes: 200, edges: 2170), indicating a decrease in microbial community complexity after DOM addition.

### Vertical variation of nitrogen/phosphorus/sulfur cycle genes and taxa in different seepage intensity regions

To understand the ecological functions of these prokaryotes in biogeochemical cycles in the Haima cold seep, we further elucidated the genes and significant taxa related to nitrogen, phosphorus, and sulfur cycles through metagenomic analysis. The results suggested that there were no discernible disparities in the primary contributors of the key genes between the active (R1 and R2) and non-active (R3, R4, and R5) zones, whether they were in the water column or sediment (Fig. S2). However, notable vertical variations were observed in the relative abundance and taxa of key genes involved in the nitrogen/phosphorus/sulfur cycles between the water columns and sediments. Proteobacteria are crucial in mediating the elemental cycles in cold seeps, with Gammaproteobacteria serving as significant mediators in the water column and sediments. Alpha- and Beta-proteobacteria primarily functioned in the water column, whereas Delta- and Epsilon-proteobacteria were mainly involved in the nitrogen, phosphorus, and sulfur cycles in near-bottom waters and sediments (Fig. S2).

**Fig 5:**
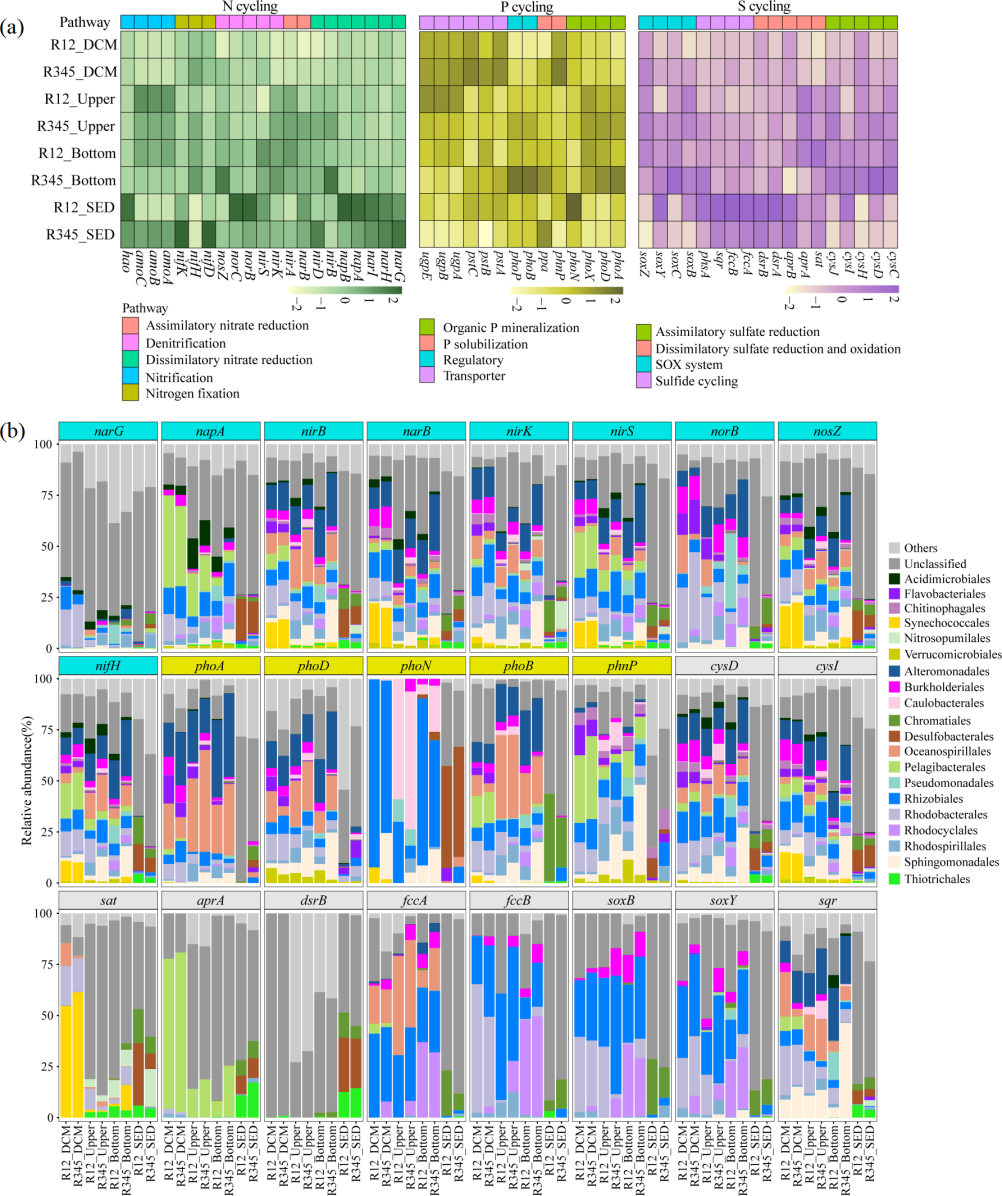
Metagenomic analysis of nitrogen/phosphorus/sulfur cycles in Haima cold seep. (**a**) The abundance value (scaled-row) of key genes involved in nitrogen/ phosphorus/sulfur cycles. (**b**) Microbial taxa (at order level) of these genes in each sample. The turquoise represens genes related to the nitrogen cycle; the pink background represents genes related to the phosphorus cycle; the gray background represents genes related to the sulfur cycle.

In the nitrogen cycle, genes associated with dissimilatory nitrate reduction (*narGHI*), denitrification (*norBC*), and nitrogen fixation (*nifDHK*) exhibited higher relative abundances in the sediment than in the water column, suggesting that these processes were more favorable in sediments with lower dissolved oxygen levels (Fig. 5a; Fig. S3). The relative abundance of nitrate reduction and denitrification was higher in active zones than in the non-active zones, whereas the relative abundance of genes related to nitrogen fixation was higher in the non-active zones (Fig. S3). The taxa of nitrogen-related genes showed similarities across different water depths; however, notable differences were observed between the water column and sediment. The major taxa mediating the nitrogen cycle in the water column included Alteromonadales, Burkholderiales, Oceanospirillales, Pelagibacterales, Rhodobacterales, Rhizobiales, Synechococcales, and Sphingomonadales, whereas Chromatiales and Desulfobacterales were predominant in the sediment (Fig. 5b).

Nine processes were identified in the phosphorus cycle. Most of these metabolic processes exhibited higher abundance in both the upper and bottom layers (Fig. S3). The relative abundance of alkaline phosphatase genes (*phoA*, *phoD,* and *phoX* genes) was high in upper and bottom layers, whereas *phoN* gene was higher in the sediment (Fig. 5a). The *phoA* and *phoD* genes were mostly associated with Alteromonadales, Burkholderiales, Oceanospirillales, Rhodobacterales, and Rhizobiales in the water column. The *phoN* gene in the water column was mainly contributed by Rhizobiales and Caulobacterales, whereas in sediment, it was predominantly mediated by Desulfobacterales (Fig. 5b).

In the sulfur cycle, the relative abundances of dissimilatory sulfate reduction and oxidation, sulfur disproportionation, sulfur oxidation, and sulfur reduction were higher in sediments, particularly in the active zone. By contrast, other processes were more prevalent in the bottom waters of the non-active zone (Fig. S3). Genes related to assimilatory sulfate reduction (e.g., *cysCDHJ*) were most abundant in the bottom layers, with higher levels observed in non-active zones than in the active zones. Conversely, genes associated with dissimilatory sulfate reduction and oxidation (such as *aprAB* and *dsrAB*), SOX system, and sulfide cycling (such as *fccAB*) were the most prevalent in sediments (Fig. 5a), with higher levels in the active zones than in non-active zones. The *cysD* and *cysI* genes were mostly associated with taxa similar to the main contributors of nitrogen-related genes. Pelagibacterales was the main contributor to dissimilatory sulfate reduction and oxidation genes (*aprA*) in the water column, whereas this gene was predominantly mediated by Chromatiales, Desulfobacterales, and Thiotrichales in the sediment. Genes associated with the SOX system and sulfide cycling in the water column were primarily mediated by Rhizobiales, Rhodobacterales, and Rhodocyclales, whereas those in sediment were also affiliated with Chromatiales, indicating a significant role of Chromatiales in the sulfur cycle in cold-seep sediment (Fig. 5b).

Given the close relationship between methane and sulfur metabolism (such as methane oxidation and sulfate reduction) (7, 8), we conducted a preliminary analysis of key functional genes in methane cycling (*mcrA* for methanogenesis and *pmoA* for methane oxidation), to identify the potential mediators involved in both sulfur and methane metabolisms. The relative abundance of methane metabolism genes was highest in sediments, with the *mcrA* gene showing the greatest abundance in sediments from non-active zones (Fig. S4). The main taxa responsible for methanogenesis (*mcrA* gene) were Methanomicrobia and other unclassified Archaea. By contrast, the *pmoA* gene was more abundant in sediments from active zones. The gene was affiliated with Betaproteobacteria in water columns and Gammaproteobacteria in sediments (Fig. S4).

### Potential interconnections between nitrogen/phosphorus/sulfur and their environmental drivers

We further explored the potential coupling relationships in the nitrogen/phosphorus/sulfur cycles at different depths and detected close correlations between these processes. For example, gene families associated with S oxidation (*soxB*, *sqr*, and *fccB*) were significantly positively correlated (*P* < 0.05) with denitrification-related genes (*nirK* and *norC*) and *nifHK* genes involved in nitrogen fixation in DCM, suggesting a close relationship between these processes (Fig. S5). Alteromonadales, Burkholderiales, Rhizobiales, Rhodocyclales, and Chromatiales collectively are the main contributors to the genes related to nitrate reduction, denitrification, and sulfur oxidation, indicating their potential importance in driving the coupling of nitrogen and sulfur cycles. To elucidate the potential environmental drivers of nitrogen, phosphorus, and sulfur cycles in cold-seep water columns, the Mantel test was used to determine the correlation between the abundance of key genes associated with nitrogen, phosphorus, and sulfur cycles and environmental variables. The results revealed that inorganic nutrients (SRP, NO_3_^−^, and DIN) were the primary factors influencing the distribution of genes related to nitrogen and phosphorus cycles, whereas cDOM was identified as a crucial factor shaping the distribution of genes related to the sulfur cycle (Fig. S6).

## DISCUSSION

### Limited impact of seepage on the prokaryotic community composition

Seepage activity accompanies substantial releases of methane and hydrogen sulfide. The fluid is continuously released into seawater and sometimes reaches 100–800 m above the seabed, which may alter the environmental characteristics of the water column (3, 6). A recent study on the Haima cold seep indicated that bubble plumes transported nutrient-rich seawater to the thermocline and that the increased surface water chlorophyll-a may be related to methane (26). However, whether the impact of seepage activity on prokaryotic communities in the water column above cold seep extends to the euphotic zone remains unclear. In this study, we found that the impact of cold seepage activity on prokaryotic community composition may be limited. In the vertical direction, the prokaryotic communities in the near-bottom water were more similar to those in the deep layer of the euphotic zone (44.60%) and at a depth of 400 m (50.89%), compared to sediment (18.00%; Fig. 3a). These results implied that prokaryotes in sediments are unlikely to be substantially transported to the overlying water column along with organic matter or bubbles released by seepage. Previous studies have investigated water depths (600, 800, and 1000 m) not covered in our study, with findings demonstrating a comparable community composition from 200 m to the bottom water (9, 10). In addition, similar vertical prokaryotic distribution patterns have been observed in non-cold-seep areas of the SCS (from the Luzon Strait to the SCS basin) and other regions, such as the Atlantic and Antarctic Peninsula (27, 28). The similarity in the composition of prokaryotic communities in mesopelagic water may be associated with sinking particles, which have been reported to act as carriers, transporting attached microorganisms into the deep sea and promoting vertical connectivity (12, 29). For example, the significant taxon Alteromonadales (with a relative abundance reaching up to 60.72%) identified in this study is a particle-associated inhabitant widely distributed in the ocean (10, 30). In the horizontal direction, the differences in prokaryotic composition between the active and non-active zones were manifested only in near-bottom water. Specifically, the relative abundance of archaea Nitrosopumilales and marine group II in the active zone were notably higher than those in the non-active zone (Fig. 2a). Nitrosopumilales dominated the water masses, exhibiting high apparent oxygen utilization and DOM, particularly humic-like components, and tended to co-occur with marine group II (31). A previous study also indicated the influence of seepage intensity on bottom-water bacterial community dynamics (6). In summary, we believe that seepage fluids may persist in the water column for several hundred meters; however, their influence on prokaryotic communities is limited to near-bottom waters. This finding, to some extent, supports the development of deep-sea cold-seep resources.

### DOM as an important factor shaping the prokaryotic community composition

Microorganisms are the primary participants in the mineralization of organic matter, utilizing various survival strategies such as copiotrophy or oligotrophy to acquire nutrients and energy from organic compounds, making them significant in marine biogeochemical cycles (32). In this study, organic matter was a major factor inﬂuencing prokaryotic community composition (Fig. 3c). We further explored the impact of different organic matter components on prokaryotic communities through *in situ* enrichment experiments and focused on the identity and dynamics of the most responsive taxonomic group at order and genus levels, that is, those that exhibited the largest changes in relative abundance at the end of experiments (33). The results demonstrated that different DOM components had varying effects on the prokaryotic communities, indicating that distinct microbial groups exhibit selective preferences for utilizing organic substances. DON addition increased the relative abundance of Rhodobacterales (Fig. 4c), suggesting a competitive advantage for Rhodobacterales in nutrient acquisition from nitrogen-containing organic matter (34). There was a notable increase in the relative abundance of Alteromonadales and Pseudomonadales after DOS addition (Fig. 4c), suggesting their potential advantage in the degradation of sulfur-containing polycyclic aromatic hydrocarbons (35). The *Vibrio* genus was a rare taxon (representing a relative abundance of <0.1% in the *in situ* community) in the original waters. However, particularly remarkable was the rapid increase in the *Vibrio* genus, from a relative abundance of 0.31%–1.07% in the CK treatments to 38.77%–68.43% at the end of the incubation in DOP treatments (Fig. 4c and d). These findings suggest that under specific environmental conditions, some bacteria of the “rare biosphere” exhibit explosive increases and become core groups, supporting the seed bank hypothesis (33, 36). Microcosm experiments in the mesopelagic North Atlantic also showed that the *Vibrio* genus increased from a relative abundance of 0.03% in the original waters to 37%–39% after organic matter (pyruvate + acetate) enrichment (37), suggesting the influence of bottom-up effects on microbial community structure. To further investigate the relationship between *Vibrio* and phosphate, a GAM analysis was conducted based on published environmental parameters from the Tara Ocean database. The results revealed that the relative abundance of *Vibrio* increased with increasing phosphate concentration (Fig. S7). Previous studies have also indicated that phosphorus (orthophosphate and total phosphorus) is a main factor that determines changes in the abundance of planktonic *Vibrio* (38, 39). In addition, this genus possesses various extracellular hydrolytic enzymes, such as alkaline phosphatasers, which release phosphate and carbon from a phosphoester (C-O-P bond) (40, 41). Moreover, many *Vibrio* species exhibit rapid growth with excessively short replication times (10 min) (42), contributing to their rapid establishment as core species in abundance (43). The *Vibrio* genus rapidly responds to DOP and affects the carbon and phosphorus cycles in marine systems (43). However, the mechanism by which this group efficiently utilizes organophosphorus compounds requires further investigation.

### Proteobacteria as the versatile player driving nitrogen/phosphorus/sulfur cycles in cold-seep water columns

Microorganisms are recognized as crucial drivers of nutrient and biogeochemical cycles in oceans and coastal waters (44). Current studies on cold-seep elemental cycles have primarily focused on carbon, nitrogen, and sulfur in sediments (5, 45, 46). However, the role of microbial communities in cold-seep water columns in biogeochemical cycles remains unclear. Our findings revealed notable differences in the primary contributors of genes involved in mediating the nitrogen, phosphorus, and sulfur cycles between the water column and sediment. In the water column, the contributors were primarily Gammaproteobacteria, Alphaproteobacteria, and Betaproteobacteria, whereas in the sediment, they were Deltaproteobacteria and Gammaproteobacteria (Fig. S2). Similarly, previous studies on cold-seep sediments have indicated that Proteobacteria are significant in the elemental cycles of carbon, nitrogen, and sulfur (45–47). Notably, most of these groups were the main contributors to multiple genes, implying that they are versatile players, potentially participating in multiple metabolic processes and driving the coupling of elemental cycles. For example, Betaproteobacteria (Burkholderiales and Rhodocyclales) and Alpahaproteobacteria (Rhizobiales) were the major contributors to genes associated with denitrification, nitrogen fixation, and sulfur oxidation in the bottom layer (Fig. 5b). Moreover, significant and positive correlations were observed between these genes, such as *nifDHK* and *fccB* (*P* < 0.05; Fig. S4a through c). These results suggested that they have the potential to couple nitrogen and sulfur cycles in cold-seep water columns. In cold-seep sediments, some sulfur-oxidizing bacteria (e.g., *Sulfurovum* and *Thiobacillus*) and sulfate-reducing bacteria (e.g., Desulfobulbales and Desulfatiglandales) have also been shown to possess diverse metabolic potential, including nitrogen fixation, nitrate reduction, and carbon fixation (47, 48). However, for a comprehensive understanding of the metabolic potential of prokaryotes in cold-seep water columns, genomic assembly and other omics technologies should be integrated for further analyses.

In addition, phosphorus is an essential element for all cells, and hydrocarbon seepage introduces abundant organic matter but litter nitrogen and phosphorus (1). Phosphorus limitation constrains marine productivity and affects trophic interactions and organic-matter cycles (49, 50). However, our knowledge of the phosphorus cycle in cold-seep environments is limited. Phosphoesters account for over 75% of the DOP compounds in oceanic waters (51). Alkaline phosphatase is a well-known enzyme that releases phosphate from phosphoesters (52). In this study, genes encoding alkaline phosphatase (*phoADX*) were primarily mediated by Alteromonadales and Oceanospirillales of the class Gammaproteobacteria and order Sphingomonadales of the class Alphaproteobacteria (Fig. 5b), highlighting the significance of these taxa in the hydrolysis of organic phosphorus, releasing biologically available phosphate. In oligotrophic regions at low latitudes, DOP concentration markedly exceeds that of inorganic phosphate, making it a potential phosphorus source for many microorganisms (53). Phosphorus and nitrogen cycles exhibited close connections, such as a significant positive correlation between genes associated with nitrogen fixation (*nifDHK*) and organic phosphorus hydrolysis (*phoADN*; Fig. S4a through c). This may be because DOP hydrolysis exacerbates nitrogen limitation, thereby expanding the ecological niche of nitrogen-fixing organisms and promoting biological nitrogen fixation (54), indicating that phosphorus is a key factor influencing nitrogen fixation. Nitrogen-fixing organisms, in turn, are crucial drivers of the phosphorus cycle in cold seeps (55). Further field investigations and additional cultivation experiments are required to better understand the microbially driven phosphorus cycle in cold seeps.

### Conclusion

**Fig 6:**
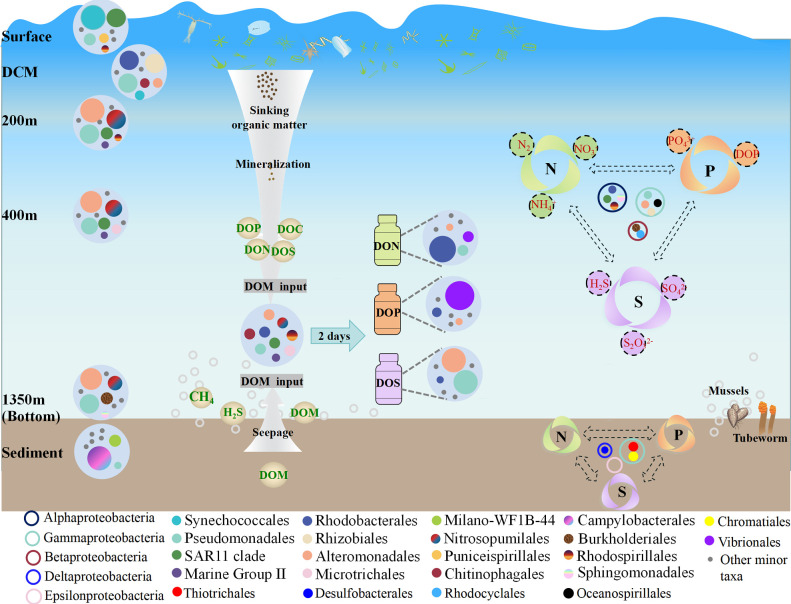
Schematic diagram showing prokaryotic community and its response to dissolved organic matter with different components, and the dominant taxa involved in biogeochemical cycling in cold seep.

In this study, the prokaryotic communities and biogeochemical cycle-associated taxa in the Haima cold seep exhibited variations in composition between water columns and sediments (Fig. 6). The vertical composition of prokaryotic communities in the near-bottom water was more similar to that of the deep layer of the euphotic zone and a depth of 400 m than that of the sediments. Horizontally, differences in prokaryotic community composition between active and non-active zones were only evident in the bottom water, with no marked differences observed in the other water layers. Field investigations and *in situ* enrichment experiments have demonstrated that organic matter is an important factor shaping prokaryotic community composition and that DOM with different components has varying effects on prokaryotic community composition. Proteobacteria was the most important group driving the cycles of nitrogen, phosphorus, and sulfur in the cold-seep ecosystem. There were no substantial differences in the primary contributors of genes between the active and non-active zones in the water column. Therefore, the impact of seepage activity on prokaryotic communities and biogeochemical cycles in the upper water column is weak, to some extent, supporting the development of deep-sea cold-seep resources. This study offers insights into the cultivation of microorganisms in extreme environments and deepens our understanding of their functional and ecological roles in biogeochemical cycles.

## MATERIALS AND METHODS

### Sample collection

**Fig 7:**
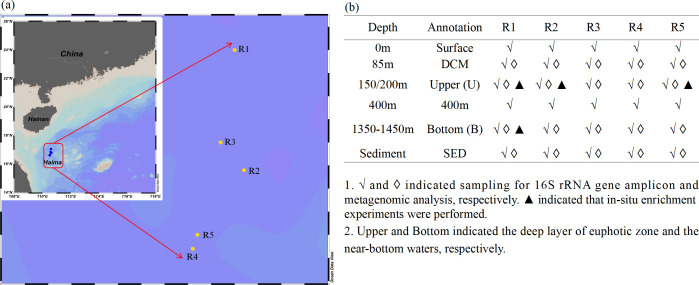
Sampling locations in the Haima cold seep on the northern slope of the South China Sea. (a)The sampling sites of R1–R5 are highlighted by yellow dots. (b) Specific sampling water depths at each site.

Samples were obtained from Haima cold seeps (R1–R5) on the northern slope of the SCS in May 2021 (Fig. 7A). The sampling depths encompassed 0, 85, 150/200, 400 m, and near-bottom layer (1,350–1,450 m; Figure 7B). Seawater samples were collected using a rosette system equipped with a CTD (conductivity-temperature-depth) sensor (Sea-Bird 911 plus, Sea-Bird Electronics, Bellevue, WA, USA). Surface sediment samples were acquired through the unmanned submersible “Haima” Remotely Operated Vehicle. *In situ* methane (CH_4_) and DO were measured using methane (METS Methane Sensor; FRANATECH, Germany) and SBE dissolved oxygen (model 43) sensors mounted on the CTD.

Based on the CH_4_ concentration and observation of *in situ* gas bubbles, R1 and R2 were classified as active zones with continuous gas bubbles emissions from the seabed and a CH_4_ concentration of approximately 80 nmol L^−1^. By contrast, R3, R4, and R5 were categorized as non-active zones with no observed gas bubbles and a CH_4_ concentration of approximately 0 nmol L^−1^. The depths of the deep chlorophyll maximum, 150/200 m, 1,350–1,450 m (near the bottom), and sediment were labeled DCM, Upper, Bottom, and SED, respectively. The metagenomic data of R1 and R2 were averaged to represent the active zones and labeled as R12-DCM/Upper/Bottom/SED. *In situ* enrichment experiments for R1 and R2 were averaged and denoted as R12-U. The results of R3, R4, and R5 were averaged to represent the non-active zones and labeled as R345-DCM/Upper/Bottom/SED.

### DOM enrichment experiments

The response of prokaryotic community composition to the addition of DOC, DON, DOP, and DOS was tested at R1 (150 m), R2 (200 m), R5 (200 m), and R1 (approximately 1,430 m; Figure 7B). Seawater was sampled in duplicate 2.4 L polycarbonate bottles. Seawater samples from the upper layer of each site in the bottles were amended with different DOM compositions as follows: CK (no DOM addition), DON (urea, 48 µM), DOP (ATP, 1 µM), and DOS (dibenzothiophene, 30 µM). In addition to the aforementioned four treatments, DOC (glucose, 200 µM) was added to the bottom water layer from R1. Seawater samples for treatments from the upper and bottom layers were incubated at approximately 16°C with screens to shade light and at 4°C in darkness, respectively. All bottles were incubated for 48 h, and seawater was collected for heterotrophic bacterial abundance and DNA extraction.

### DNA extraction, PCR amplicon, sequencing, and analysis

Genomic DNA was extracted using the E. Z. N. A Water DNA kit (OMEGA, USA). The bacterial 16S rRNA gene V3–V4 region was amplified using the specific primers 341F (5′-CCTAYGGGRBGCASCAG-3′) and 806R (5′-GGACTACNNGGGTATCTAAT-3′). Sequencing was performed by Biozeron Biological Technology Co. Ltd. (Shanghai, China) on a MiSeq platform (Illumina, San Diego, CA, USA). Amplicon sequence variants (ASVs) were generated using the divisive amplicon denoising algorithm in QIIME2. The ASV matrices were rarefied using the minimum sample size for bacteria. Taxonomy annotation was performed based on the naive Bayes classifier in QIIME2 using the SILVA database (v. 138.1) with a confidence threshold of 80%.

### Metagenomic analysis

Metagenomic reads were subjected to quality trimming using Trimmomatic to remove low-quality reads. The taxonomy of clean reads for each sample was determined using Kraken2 (56) with a customized Kraken database. The abundance of taxa was estimated using the Bracken software (https://ccb.jhu.edu/software/bracken/). Clean sequence reads were generated into a set of contigs for each sample using MegaHit with “--min-contig-len 500” parameters (57). The open reading frames of the assembled contigs were predicted using Prodigal (v2.6.3) (58), and all open reading frames were generated as a set of unique genes after clustering using CD-HIT at 95% identity and 90% coverage (59). The longest sequence of each cluster was considered the representative sequence of each gene in the unique gene set. To calculate gene abundance within the total samples, Salmon software (60) was used to determine the read number for each gene. The predicted gene fragments were searched against KEGG (https://www.genome.jp/kegg/pathway.html), NCycDB (61), PCycDB (62), SCycDB (63), and MCycDB (64) reference databases using DIAMOND for functional annotation.

### Statistical analysis

The alpha diversity indices were calculated with the “Picante” package in R. The “lm” function in R was used to analyze the correlation between alpha diversity indicesand depth, using a linear regression model. Beta diversity was estimated using principal coordinate analysis (PCoA) based on the Bray–Curtis distance. Permutational multivariate analysis of variance was performed using the R “vegan” package to assess the significance of compositional differences between sampling depths. SIMPER analysis was conducted using PRIMER 5.0 to calculate the similarity of community composition at different depths based on Bray–Curtis similarity. Spearman analysis was performed using the “ggpmisc” package in R to estimate the correlation between key genes associated with nitrogen, phosphorus, and sulfur cycles. Redundancy analysis (RDA) was conducted using the R “vegan” package to evaluate the relationships between key microbial taxa (20 top relative abundance) and environmental variables (log-transformed). A generalized additive model (GAM) was constructed to explore the relationship between the relative abundance of the *Vibrio* genus and phosphate concentration via the “geom_smooth” function of the “ggplot2” package.

## Data Availability

All raw sequences in this study were deposited in the NCBI Sequence Read Archive under BioProject ID PRJNA1103165 and PRJNA944722.

## SUPPLEMENTAL MATERIAL

Supplemental material msphere.00549-24-s0001.docx Supplemental materials and methods and Fig. S1 to S7.

ASM does not own the copyrights to Supplemental Material that may be linked to, or accessed through, an article. The authors have granted ASM a non-exclusive, world-wide license to publish the Supplemental Material files. Please contact the corresponding author directly for reuse.

## References

[1] Joye SB. 2020. The geology and biogeochemistry of hydrocarbon seeps. Annu Rev Earth Planet Sci 48:205–231. doi:10.1146/annurev-earth-063016-020052

[2] Feng D, Peckmann J, Li N, Kiel S, Qiu J, Liang Q, Carney RS, Peng Y, Tao J, Chen D. 2018. The stable isotope fingerprint of chemosymbiosis in the shell organic matrix of seep-dwelling bivalves. Chem Geol 479:241–250. doi:10.1016/j.chemgeo.2018.01.015

[3] Bayon G, Birot D, Ruffine L, Caprais JC, Ponzevera E, Bollinger C, Donval JP, Charlou JL, Voisset M, Grimaud S. 2011. Evidence for intense REE scavenging at cold seeps from the Niger Delta margin. Earth Planet Sci Lett 312:443–452. doi:10.1016/j.epsl.2011.10.008

[4] Sisma-Ventura G, Bialik OM, Makovsky Y, Rahav E, Ozer T, Kanari M, Marmen S, Belkin N, Guy-Haim T, Antler G, Herut B, Rubin-Blum M. 2022. Cold seeps alter the near-bottom biogeochemistry in the ultraoligotrophic Southeastern Mediterranean sea. Deep Sea Res Part I: Oceanogr Res Pap 183:103744. doi:10.1016/j.dsr.2022.103744

[5] Chen Y, Dai T, Li N, Li Q, Lyu Y, Di P, Lyu L, Zhang S, Li J. 2023. Environmental heterogeneity shapes the C and S cycling-associated microbial community in Haima’S cold seeps. Front Microbiol 14:1199853. doi:10.3389/fmicb.2023.1199853 37502402 PMC10370420

[6] Li X, Dai Z, Di P, Feng J, Tao J, Chen D, Li N, Li Y. 2021. Distinct bottom-water bacterial communities at methane seeps with various seepage intensities in Haima, South China Sea. Front Mar Sci 8:753952. doi:10.3389/fmars.2021.753952

[7] Niu M, Fan X, Zhuang G, Liang Q, Wang F. 2017. Methane-metabolizing microbial communities in sediments of the Haima cold seep area, northwest slope of the South China Sea. FEMS Microbiol Ecol 93:fix101. doi:10.1093/femsec/fix101 28934399

[8] Scheller S, Yu H, Chadwick GL, McGlynn SE, Orphan VJ. 2016. Artificial electron acceptors decouple archaeal methane oxidation from sulfate reduction. Science 351:703–707. doi:10.1126/science.aad7154 26912857

[9] Huang Y, Feng J, Kong J, Sun L, Zhang M, Huang Y, Tang L, Zhang S, Yang Z. 2023. Community assemblages and species coexistence of prokaryotes controlled by local environmental heterogeneity in a cold seep water column. Sci Total Environ 868:161725. doi:10.1016/j.scitotenv.2023.161725 36669671

[10] Zhang Y, Jing H, Peng X. 2020. Vertical shifts of particle-attached and free-living prokaryotes in the water column above the cold seeps of the South China sea. Mar Pollut Bull 156:111230. doi:10.1016/j.marpolbul.2020.111230 32510376

[11] Wu Y, Qiu J, Qian P, Wang Y. 2018. The vertical distribution of prokaryotes in the surface sediment of Jiaolong cold seep at the northern South China Sea. Extremophiles 22:499–510. doi:10.1007/s00792-018-1012-0 29442249

[12] Mestre M, Ruiz-González C, Logares R, Duarte CM, Gasol JM, Sala MM. 2018. Sinking particles promote vertical connectivity in the ocean microbiome. Proc Natl Acad Sci USA 115:E6799–E6807. doi:10.1073/pnas.1802470115 29967136 PMC6055141

[13] Sebastián M, Forn I, Auladell A, Gómez-Letona M, Sala MM, Gasol JM, Marrasé C. 2021. Differential recruitment of opportunistic taxa leads to contrasting abilities in carbon processing by bathypelagic and surface microbial communities. Environ Microbiol 23:190–206. doi:10.1111/1462-2920.15292 33089653

[14] Giering SLC, Sanders R, Lampitt RS, Anderson TR, Tamburini C, Boutrif M, Zubkov MV, Marsay CM, Henson SA, Saw K, Cook K, Mayor DJ. 2014. Reconciliation of the carbon budget in the ocean’s twilight zone. Nature 507:480–483. doi:10.1038/nature13123 24670767

[15] Ke Z, Li R, Chen Y, Chen D, Chen Z, Lian X, Tan Y. 2022. A preliminary study of macrofaunal communities and their carbon and nitrogen stable isotopes in the Haima cold seeps, South China Sea. Deep Sea Res Part I: Oceanogr Res Pap 184:103774. doi:10.1016/j.dsr.2022.103774

[16] Sert MF, D’Andrilli J, Gründger F, Niemann H, Granskog MA, Pavlov AK, Ferré B, Silyakova A. 2020. Compositional differences in dissolved organic matter between arctic cold seeps versus non-seep sites at the svalbard continental margin and the Barents Sea. Front Earth Sci 8:552731. doi:10.3389/feart.2020.552731

[17] Hu T, Luo M, Qi Y, He D, Chen L, Xu Y, Chen D. 2023. Molecular evidence for the production of labile, sulfur-bearing dissolved organic matter in the seep sediments of the South China sea. Water Res 233:119732. doi:10.1016/j.watres.2023.119732 36801578

[18] Grabowski E, Letelier RM, Laws EA, Karl DM. 2019. Coupling carbon and energy fluxes in the North Pacific subtropical gyre. Nat Commun 10:1895. doi:10.1038/s41467-019-09772-z 31028256 PMC6486601

[19] Liu R, Wang L, Liu Q, Wang Z, Li Z, Fang J, Zhang L, Luo M. 2018. Depth-resolved distribution of particle-attached and free-living bacterial communities in the water column of the New Britain Trench. Front Microbiol 9:625. doi:10.3389/fmicb.2018.00625 29670597 PMC5893722

[20] Teeling H, Fuchs BM, Becher D, Klockow C, Gardebrecht A, Bennke CM, Kassabgy M, Huang S, Mann AJ, Waldmann J, et al.. 2012. Substrate-controlled succession of marine bacterioplankton populations induced by a phytoplankton bloom. Science 336:608–611. doi:10.1126/science.1218344 22556258

[21] Needham DM, Fuhrman JA. 2016. Pronounced daily succession of phytoplankton, archaea and bacteria following a spring bloom. Nat Microbiol 1:16005. doi:10.1038/nmicrobiol.2016.5 27572439

[22] Moran MA, Durham BP. 2019. Sulfur metabolites in the pelagic ocean. Nat Rev Microbiol 17:665–678. doi:10.1038/s41579-019-0250-1 31485034

[23] Moran MA, Belas R, Schell MA, González JM, Sun F, Sun S, Binder BJ, Edmonds J, Ye W, Orcutt B, Howard EC, Meile C, Palefsky W, Goesmann A, Ren Q, Paulsen I, Ulrich LE, Thompson LS, Saunders E, Buchan A. 2007. Ecological genomics of marine Roseobacters. Appl Environ Microbiol 73:4559–4569. doi:10.1128/AEM.02580-06 17526795 PMC1932822

[24] Pontiller B, Martínez-García S, Joglar V, Amnebrink D, Pérez-Martínez C, González JM, Lundin D, Fernández E, Teira E, Pinhassi J. 2022. Rapid bacterioplankton transcription cascades regulate organic matter utilization during phytoplankton bloom progression in a coastal upwelling system. ISME J 16:2360–2372. doi:10.1038/s41396-022-01273-0 35804052 PMC9478159

[25] Buchan A, LeCleir GR, Gulvik CA, González JM. 2014. Master recyclers: features and functions of bacteria associated with phytoplankton blooms. Nat Rev Microbiol 12:686–698. doi:10.1038/nrmicro3326 25134618

[26] Di P, Li N, Chen L, Feng J, Chen D. 2023. Elevated nutrients and surface chlorophyll-α associated with natural methane seeps in the Haima cold seep area of the Qiongdongnan Basin, northern South China Sea. Mar Pollut Bull 191:114873. doi:10.1016/j.marpolbul.2023.114873 37031642

[27] Zhang Y, Zhao Z, Dai M, Jiao N, Herndl GJ. 2014. Drivers shaping the diversity and biogeography of total and active bacterial communities in the South China Sea. Mol Ecol 23:2260–2274. doi:10.1111/mec.12739 24684298 PMC4230472

[28] Signori CN, Thomas F, Enrich-Prast A, Pollery RCG, Sievert SM. 2014. Microbial diversity and community structure across environmental gradients in Bransfield Strait, Western Antarctic Peninsula. Front Microbiol 5:647. doi:10.3389/fmicb.2014.00647 25566198 PMC4267279

[29] Sanz-Sáez I, Sánchez P, Salazar G, Sunagawa S, de Vargas C, Bowler C, Sullivan MB, Wincker P, Karsenti E, Pedrós-Alió C, Agustí S, Gojobori T, Duarte CM, Gasol JM, Sánchez O, Acinas SG. 2023. Top abundant deep ocean heterotrophic bacteria can be retrieved by cultivation. ISME Commun 3:92. doi:10.1038/s43705-023-00290-0 37660234 PMC10475052

[30] McCarren J, Becker JW, Repeta DJ, Shi Y, Young CR, Malmstrom RR, Chisholm SW, DeLong EF. 2010. Microbial community transcriptomes reveal microbes and metabolic pathways associated with dissolved organic matter turnover in the sea. Proc Natl Acad Sci USA 107:16420–16427. doi:10.1073/pnas.1010732107 20807744 PMC2944720

[31] Gómez-Letona M, Arístegui J, Hernández-Hernández N, Álvarez-Salgado XA, Álvarez M, Delgadillo E, Pérez-Lorenzo M, Teira E, Hernández-León S, Sebastián M. 2022. Deep ocean prokaryotes and fluorescent dissolved organic matter reflect the history of the water masses across the Atlantic Ocean. Prog Oceanogr 205:102819. doi:10.1016/j.pocean.2022.102819

[32] Noell SE, Brennan E, Washburn Q, Davis EW, Hellweger FL, Giovannoni SJ. 2023. Differences in the regulatory strategies of marine oligotrophs and copiotrophs reflect differences in motility. Environ Microbiol 25:1265–1280. doi:10.1111/1462-2920.16357 36826469

[33] Sebastián M, Auguet J-C, Restrepo-Ortiz CX, Sala MM, Marrasé C, Gasol JM. 2018. Deep ocean prokaryotic communities are remarkably malleable when facing long-term starvation. Environ Microbiol 20:713–723. doi:10.1111/1462-2920.14002 29159926

[34] Zhao Z, Baltar F, Herndl GJ. 2020. Linking extracellular enzymes to phylogeny indicates a predominantly particle-associated lifestyle of deep-sea prokaryotes. Sci Adv 6:eaaz4354. doi:10.1126/sciadv.aaz4354 32494615 PMC7159927

[35] Seo J-S, Keum Y-S, Li QX. 2009. Bacterial degradation of aromatic compounds. Int J Environ Res Public Health 6:278–309. doi:10.3390/ijerph6010278 19440284 PMC2672333

[36] Pedrós-Alió C. 2006. Marine microbial diversity: can it be determined? Trends Microbiol 14:257–263. doi:10.1016/j.tim.2006.04.007 16679014

[37] Baltar F, Lundin D, Palovaara J, Lekunberri I, Reinthaler T, Herndl GJ, Pinhassi J. 2016. Prokaryotic responses to ammonium and organic carbon reveal lternative CO_2_ fixation pathways and importance of alkaline phosphatase in the mesopelagic North Atlantic. Front Microbiol 7:1670. doi:10.3389/fmicb.2016.01670 27818655 PMC5073097

[38] Gregoracci GB, Nascimento JR, Cabral AS, Paranhos R, Valentin JL, Thompson CC, Thompson FL. 2012. Structuring of bacterioplankton diversity in a large tropical bay. PLoS ONE 7:e31408. doi:10.1371/journal.pone.0031408 22363639 PMC3283626

[39] Kopprio GA, Streitenberger ME, Okuno K, Baldini M, Biancalana F, Fricke A, Martínez A, Neogi SB, Koch BP, Yamasaki S, Lara RJ. 2017. Biogeochemical and hydrological drivers of the dynamics of Vibrio species in two Patagonian estuaries. Sci Total Environ 579:646–656. doi:10.1016/j.scitotenv.2016.11.045 27871750

[40] Sebastian M, Ammerman JW. 2009. The alkaline phosphatase PhoX is more widely distributed in marine bacteria than the classical PhoA. ISME J 3:563–572. doi:10.1038/ismej.2009.10 19212430

[41] Liu J, Ding X, Xia X, Zhou L, Liu W, Lai Y, Ke Z, Tan Y. 2024. Dissolved organic phosphorus promotes Cyclotella growth and adaptability in eutrophic tropical estuaries. Appl Environ Microbiol 90:e0163723. doi:10.1128/aem.01637-23 38112726 PMC10807451

[42] Joseph SW, Colwell RR, Kaper JB. 1982. Vibrio parahaemolyticus and related halophilic vibrios. Crit Rev Microbiol 10:77–124. doi:10.3109/10408418209113506 6756788

[43] Zhang X, Lin H, Wang X, Austin B. 2018. Significance of Vibrio species in the marine organic carbon cycle—a review. Sci China Earth Sci 61:1357–1368. doi:10.1007/s11430-017-9229-x

[44] Kirchman D. 2008. Microbial ecology of the oceans. J Exp Mar Biol Ecol 269:251–252. doi:10.1016/S0022-0981(02)00027-8

[45] Chen Y, Lyu Y, Zhang J, Li Q, Lyu L, Zhou Y, Kong J, Zeng X, Zhang S, Li J. 2023. Riddles of lost city: chemotrophic prokaryotes drives carbon, sulfur, and nitrogen cycling at an extinct cold seep, South China Sea. Microbiol Spectr 11:e03338-22. doi:10.1128/spectrum.03338-22 36511717 PMC9927161

[46] Jing H, Wang R, Jiang Q, Zhang Y, Peng X. 2020. Anaerobic methane oxidation coupled to denitrification is an important potential methane sink in deep-sea cold seeps. Sci Total Environ 748:142459. doi:10.1016/j.scitotenv.2020.142459 33113688

[47] Li W, Dong X, Lu R, Zhou Y, Zheng P, Feng D, Wang Y. 2021. Microbial ecology of sulfur cycling near the sulfate-methane transition of deep-sea cold seep sediments. Environ Microbiol 23:6844–6858. doi:10.1111/1462-2920.15796 34622529

[48] Sun Q, Zhang J, Wang M, Cao L, Du Z, Sun Y, Liu S, Li C, Sun L. 2020. High-throughput sequencing reveals a potentially novel Sulfurovum species dominating the microbial communities of the seawater-sediment interface of a deep-sea cold seep in South China sea. Microorganisms 8:687. doi:10.3390/microorganisms8050687 32397229 PMC7284658

[49] Thingstad TF, Krom MD, Mantoura RFC, Flaten GAF, Groom S, Herut B, Kress N, Law CS, Pasternak A, Pitta P, Psarra S, Rassoulzadegan F, Tanaka T, Tselepides A, Wassmann P, Woodward EMS, Riser CW, Zodiatis G, Zohary T. 2005. Nature of phosphorus limitation in the ultraoligotrophic eastern Mediterranean. Science 309:1068–1071. doi:10.1126/science.1112632 16099984

[50] Yuan Z, Browning TJ, Zhang R, Wang C, Du C, Wang Y, Chen Y, Liu Z, Liu X, Shi D, Dai M. 2023. Potential drivers and consequences of regional phosphate depletion in the western subtropical North Pacific. Limnol Oceanogr Lett 8:509–518. doi:10.1002/lol2.10314

[51] Kolowith LC, Ingall ED, Benner R. 2001. Composition and cycling of marine organic phosphorus. Limnol Oceanogr 46:309–320. doi:10.4319/lo.2001.46.2.0309

[52] Shoemaker KM, McCliment EA, Moisander PH. 2020. Copepod-associated gammaproteobacterial alkaline phosphatases in the North Atlantic subtropical gyre. Front Microbiol 11:1033. doi:10.3389/fmicb.2020.01033 32523576 PMC7261903

[53] Duhamel S, Diaz JM, Adams JC, Djaoudi K, Steck V, Waggoner EM. 2021. Phosphorus as an integral component of global marine biogeochemistry. Nat Geosci 14:359–368. doi:10.1038/s41561-021-00755-8

[54] Luo Y, Lima ID, Karl DM, Doney SC. 2013. Data-based assessment of environmental controls on global marine nitrogen fixation. Biogeochemistry. doi:10.5194/bgd-10-7367-2013

[55] Yamaguchi T, Sato M, Hashihama F, Ehama M, Shiozaki T, Takahashi K, Furuya K. 2019. Basin‐scale variations in Labile dissolved phosphoric monoesters and diesters in the central North Pacific Ocean. J Geophys Res: Oceans 124:3058–3072. doi:10.1029/2018JC014763

[56] Wood DE, Salzberg SL. 2014. Kraken: ultrafast metagenomic sequence classification using exact alignments. Genome Biol 15:R46. doi:10.1186/gb-2014-15-3-r46 24580807 PMC4053813

[57] Li D, Liu C-M, Luo R, Sadakane K, Lam T-W. 2015. MEGAHIT: an ultra-fast single-node solution for large and complex metagenomics assembly via succinct de Bruijn graph. Bioinformatics 31:1674–1676. doi:10.1093/bioinformatics/btv033 25609793

[58] Hyatt D, Chen G, Locascio PF, Land ML, Larimer FW, Hauser LJ. 2010. Prodigal: prokaryotic gene recognition and translation initiation site identification. BMC Bioinformatics 11:119. doi:10.1186/1471-2105-11-119 20211023 PMC2848648

[59] Fu L, Niu B, Zhu Z, Wu S, Li W. 2012. CD-HIT: accelerated for clustering the next-generation sequencing data. Bioinformatics 28:3150–3152. doi:10.1093/bioinformatics/bts565 23060610 PMC3516142

[60] Patro R, Duggal G, Love MI, Irizarry RA, Kingsford C. 2017. Salmon provides fast and bias-aware quantification of transcript expression. Nat Methods 14:417–419. doi:10.1038/nmeth.4197 28263959 PMC5600148

[61] Tu Q, Lin L, Cheng L, Deng Y, He Z, Wren J. 2019. NCycDB: a curated integrative database for fast and accurate metagenomic profiling of nitrogen cycling genes. Bioinformatics 35:1040–1048. doi:10.1093/bioinformatics/bty741 30165481

[62] Zeng J, Tu Q, Yu X, Qian L, Wang C, Shu L, Liu F, Liu S, Huang Z, He J, Yan Q, He Z. 2022. PCycDB: a comprehensive and accurate database for fast analysis of phosphorus cycling genes. Microbiome 10:101. doi:10.1186/s40168-022-01292-1 35787295 PMC9252087

[63] Yu X, Zhou J, Song W, Xu M, He Q, Peng Y, Tian Y, Wang C, Shu L, Wang S, Yan Q, Liu J, Tu Q, He Z. 2021. SCycDB: a curated functional gene database for metagenomic profiling of sulphur cycling pathways. Mol Ecol Resour 21:924–940. doi:10.1111/1755-0998.13306

[64] Qian L, Yu X, Zhou J, Gu H, Ding J, Peng Y, He Q, Tian Y, Liu J, Wang S, Wang C, Shu L, Yan Q, He J, Liu G, Tu Q, He Z. 2022. MCycDB: a curated database for comprehensively profiling methane cycling processes of environmental microbiomes. Mol Ecol Resour 22:1803–1823. doi:10.1111/1755-0998.13589 35080120

