# Supplementary material for: Insights into prokaryotic communities and their potential functions in biogeochemical cycles in cold seep
Source: mSphere. 2024 Sep 13;9(10):e00549-24. doi: 10.1128/msphere.00549-24 (PMC11524163; doi:10.1128/msphere.00549-24)
Supplement: Supplemental material — s and methods and Fig. S1 to S7. [file msphere.00549-24-s0001.docx]

**Supporting Information for**

**Insights into prokaryotic community and its potential functions in biogeochemical cycles in cold seep**

Qiumei Quan^1, 2^, Jiaxing Liu^1^, Chaolun Li^1, 2^, Zhixin Ke^1^, Yehui Tan^1, 2 *^

^1^South China Sea Institute of Oceanology, Chinese Academy of Sciences, Guangzhou 510301, China

^2^University of Chinese Academy of Sciences, Beijing 100049, China

* Corresponding author: Yehui Tan

E-mail: tanyh@scsio.ac.cn.

Present address: South China Sea Institute of Oceanology, Chinese Academy of Sciences, Guangzhou 510301, China.

**The supplementary information includes:**

- Materials and methods
- 7 figures (Fig. S1 to S7)

**Materials and methods**

**Nutrient measurements**

In the laboratory, nitrate (NO_3_^-^), nitrite (NO_2_^-^), and ammonium (NH_4_^+^) were quantified using a flow injection analyzer (QuichChem8500; Lachat Inc., Loveland, CO, USA) following standard colorimetric methods (1). The soluble reactive phosphate (SRP) was determined using the standard molybdenum blue method with a spectrophotometer. Total dissolved nitrogen (TDN) and total dissolved phosphorus (TDP) were prepared for measurement through digestion with a K_2_S_2_O_8_ solution at 120°C for 30 min and analyzed using a flow injection analyzer (1). The TDN and TDP standards were processed along with the water samples. Dissolved inorganic nitrogen (DIN) was quantified as the sum of NO_3_^-^, NO_2_^-^, and NH_4_^+^ concentrations. The concentration of dissolved organic nitrogen (DON) was derived from the difference between TDN and DIN. Similarly, the concentration of dissolved organic phosphorus (DOP) was calculated as the difference between TDP and SRP.

**Chromophoric dissolved organic matter** **measurement**

The excitation-emission matrix fluorescence spectra of the chromophoric dissolved organic matter (cDOM) were measured using a Hitachi F-4600 fluorescence spectrometer (Hitachi High-Technologies, Tokyo, Japan), and the fluorescence intensity at excitation and emission wavelength of 350 and 450 nm, respectively, was used to assess the relative content of cDOM in water samples (2).

**Heterotrophic bacteria abundance**

The abundance of heterotrophic bacteria was determined using a flow cytometer (Becton-Dickinson Accuri C6) equipped with a laser emitting at 488 nm. All samples (200 μL) were stained with 2 μL diluted SYBR Green I (1:100 dilution of the commercial stock). The samples were carried out for 20 min in the dark at room temperature (24 °C). Stained heterotrophic bacterial cells were detected according to side light scatter and green fluorescence (530±30 nm).

**Networks analysis**

Co-occurrence networks were constructed to explore potential interactions between different microbial taxa, and how these patterns might change after adding DOM with different components. Only ASVs with a relative abundance of >0.05% across all samples were used for the analyses. Network analysis was performed based on Spearman's coefficients (*r*) matrix of the genus using the "Hmisc" R package. Spearman's correlations were filtered using robust (|*r*| > 0.60) and statistically significant correlations (BH-adjusted P <0.01) (3). To characterize the topological structure of the networks, various network topological properties were calculated using the "igraph" package in R. The constructed networks were visualized using the Gephi software (0.9.2) with an undirected network, Fruchterman-Reingold layout, and modularity class partition.

**Figure**
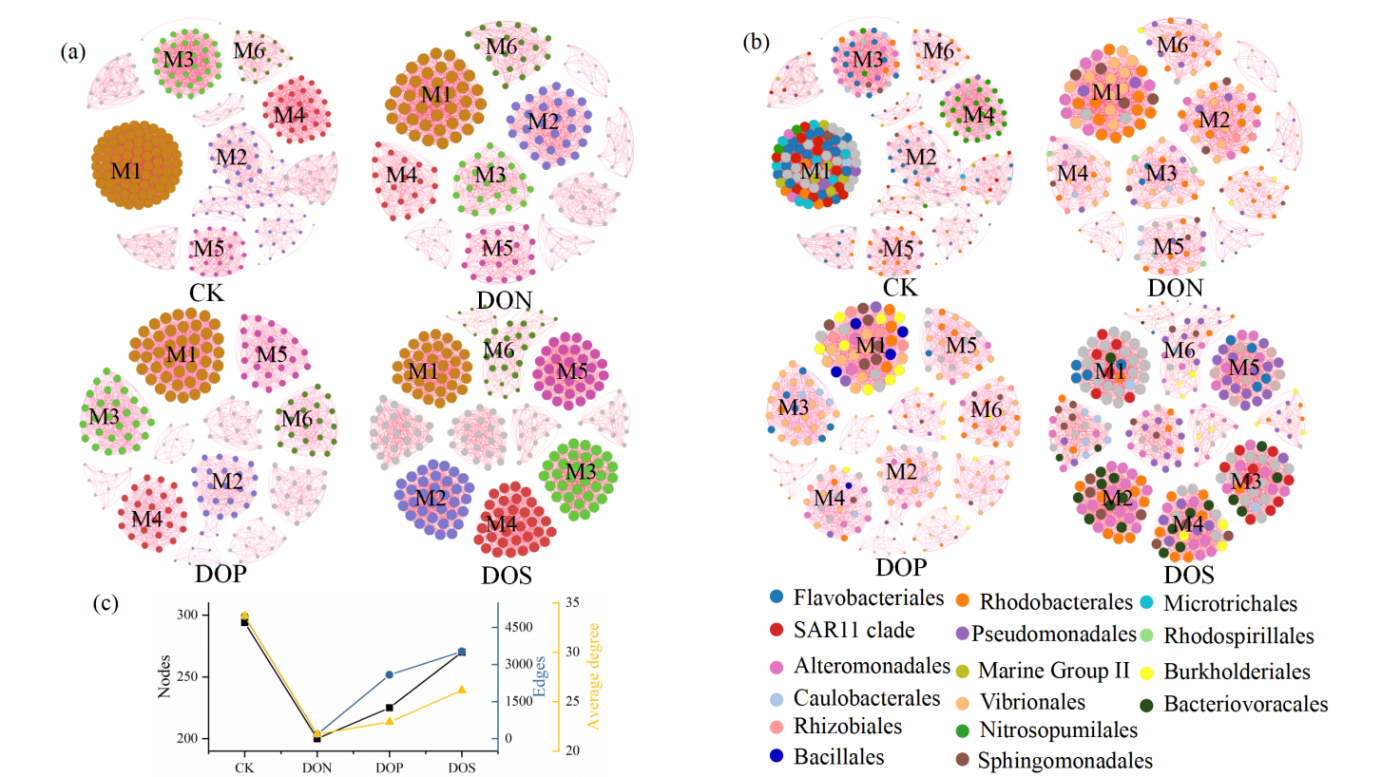


Fig.S1. Co-occurrence patterns in prokaryotic community. Networks analysis of the prokaryotic community in CK, DON, DOP, and DOS treatments. Each node represents an (a) ASVs and (b) a microorganism at the order level. (c) Topological properties (nodes, edges, and average degree) of each co-occurrence networks. Node size represents the degree of each nodes. Positive links are indicated in red and negative links are indicated in green. ASVs with relative abundances of >0.05% were selected. DON, DOP, and DOS: dissolved organic matter containing N, P, and S, respectively; CK, no addition of dissolved organic matter.


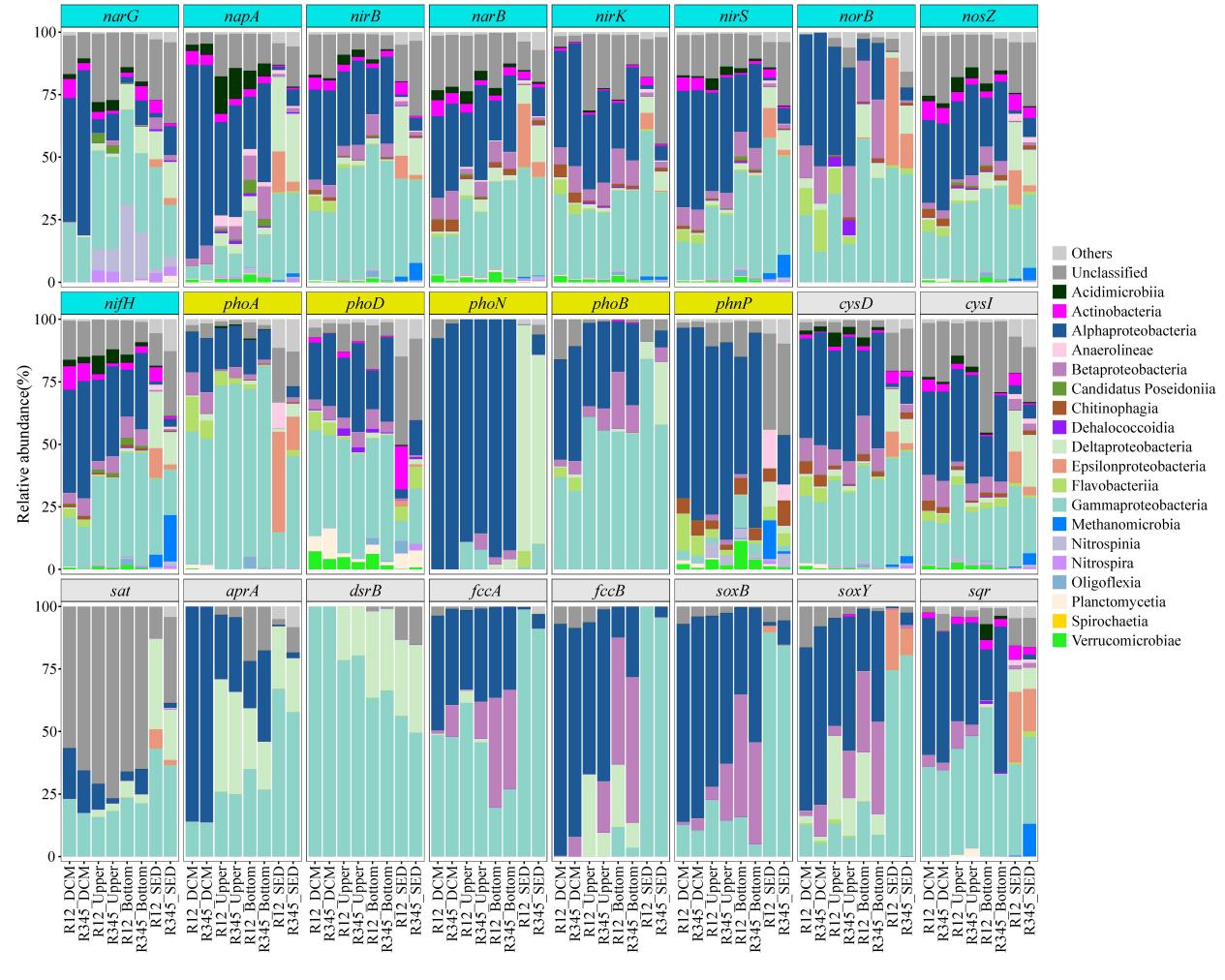


Fig. S2. Metagenomic analysis of nitrogen/phosphorus/sulfur cycle in Haima cold seep. Microbial taxa (at class level) of these genes in each sample. The turquoise represented genes related to nitrogen cycle; the pink background represents genes related to phosphorus cycle; the gray background represents genes related to sulfur cycle.


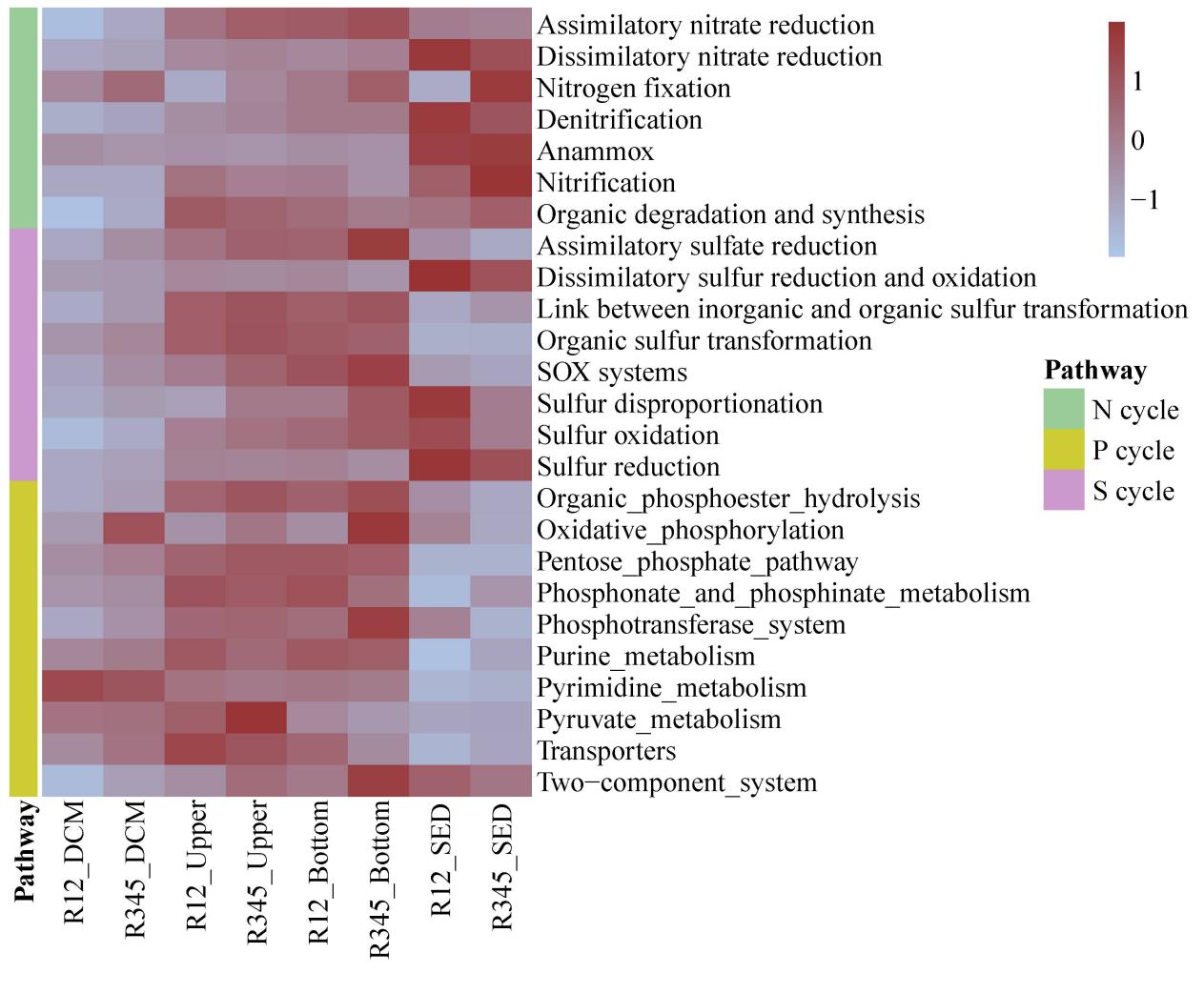


Fig. S3. The relative abundance of nitrogen/phosphorus/sulfur cycling processes in Haima cold seep based on NCycDB/PCycDB/SCycDB Database.


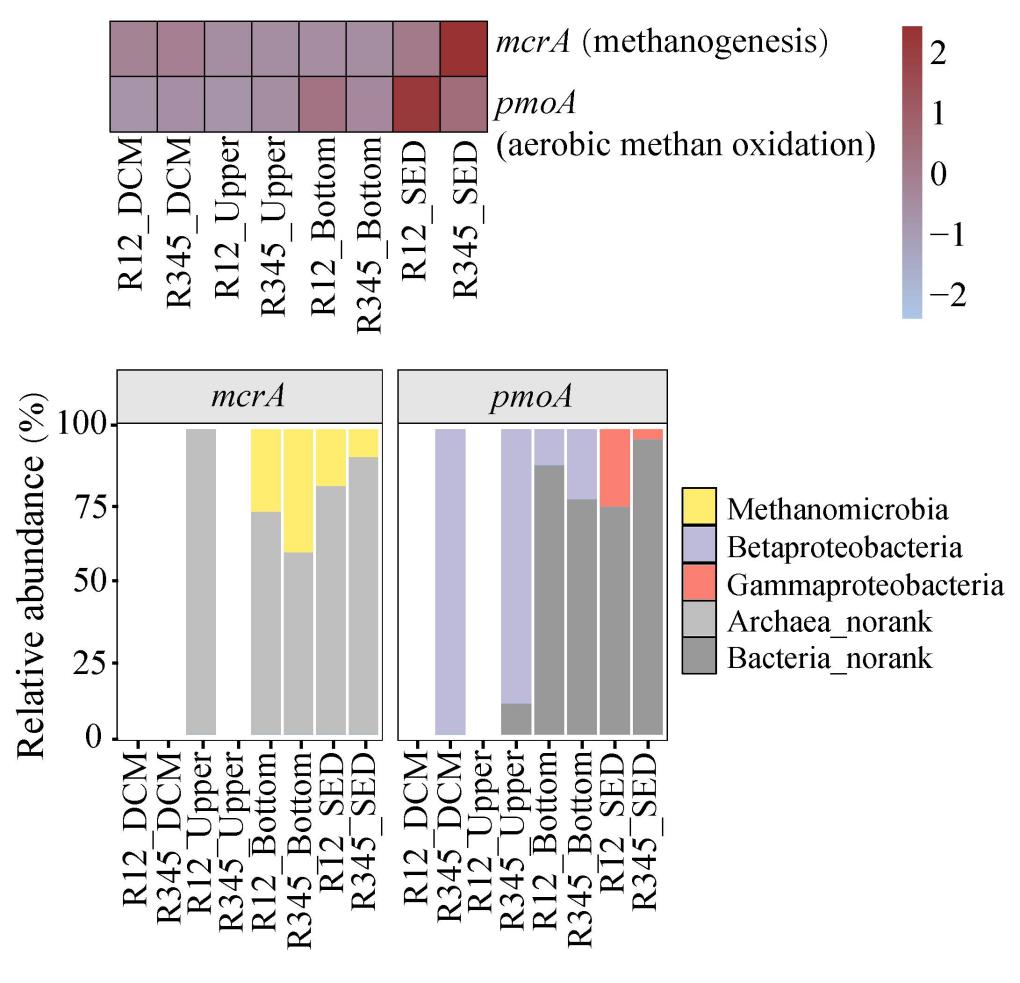


Fig. S4. The key genes involved in methane cycling in the Haima cold seep and their main contributors.


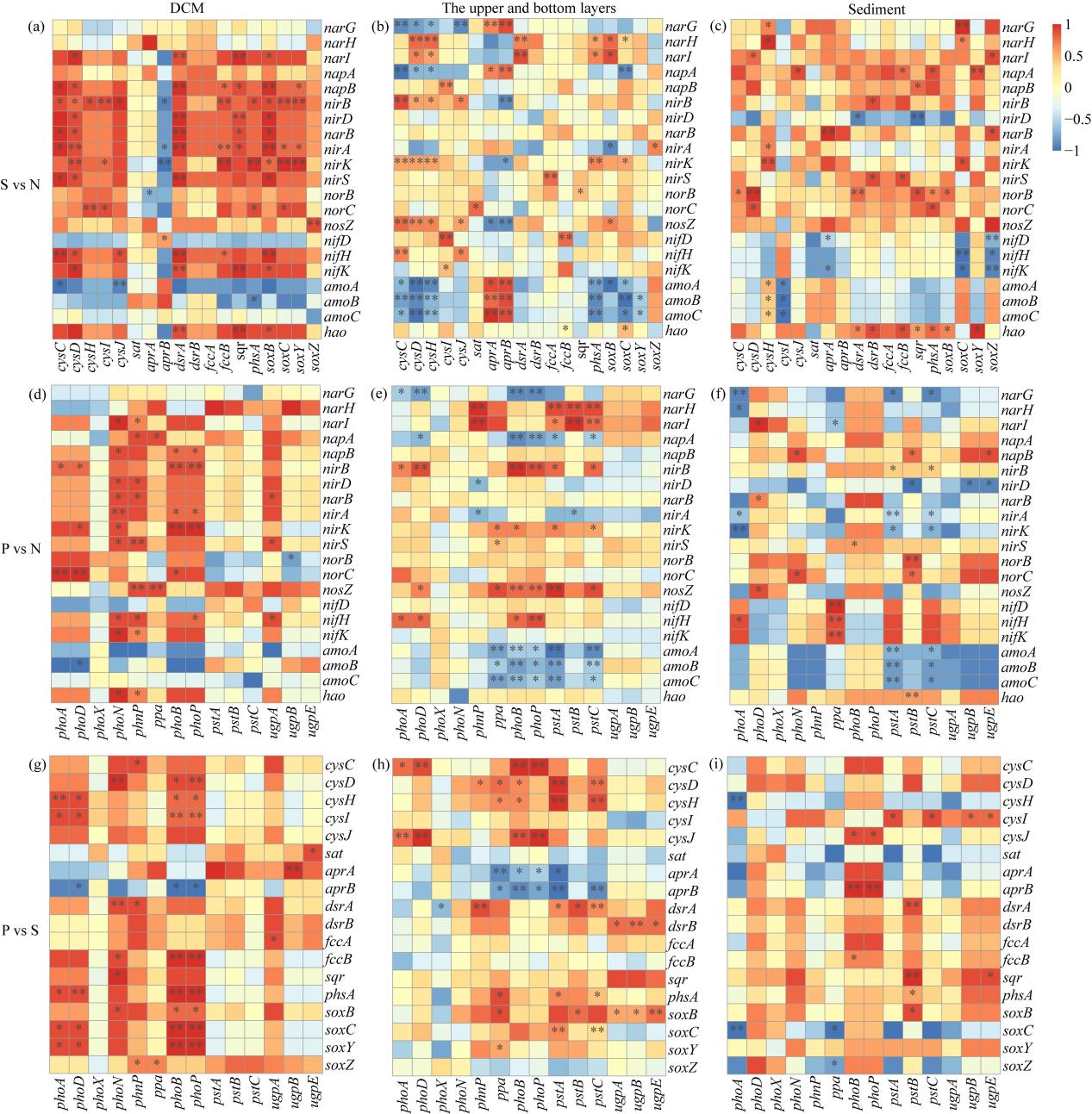


Fig. S5. Correlations between the relative abundance of key gene families. Spearman analysis revealed the correlations between key gene families involved in N/P/S cycling. (a-c) N and S cycling; (d-f) N and P cycling; and (g-i) P and S cycling. ***: P < 0.001; **: 0.001 < P < 0.01; *: 0.01< P < 0.05.


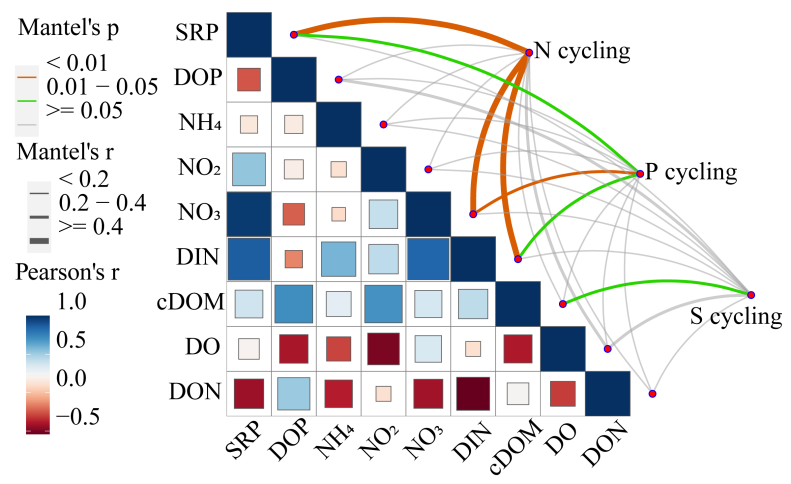


Fig. S6. Mantel tests revealed the correlationr between environmental factors (log-transformed) and nitrogen/phosphorus/sulfur cycling gene (log-transformed).


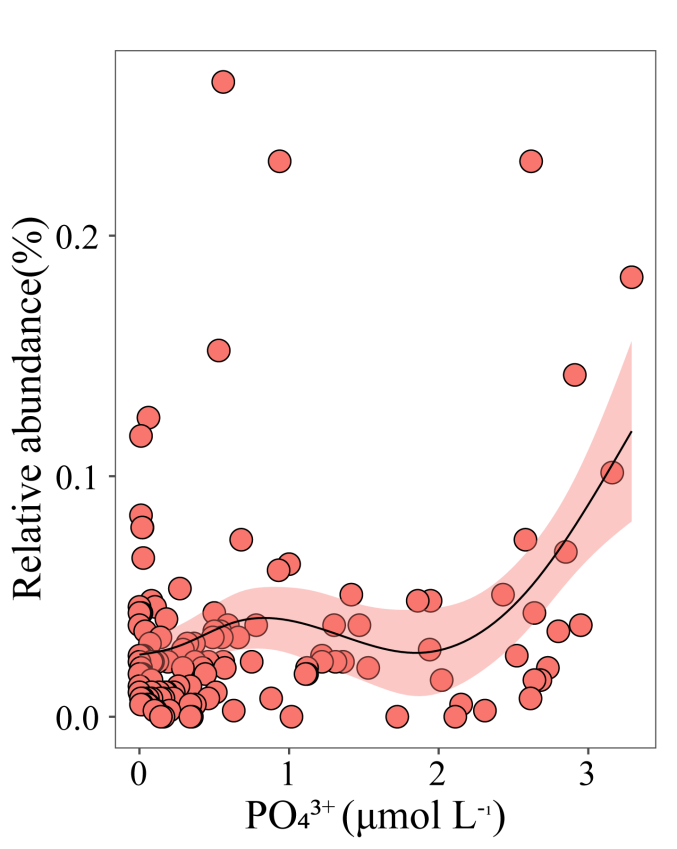


Fig. S7. GAM analysis revealing the distribution trend of *Vibrio* genus with phosphate (Data of relative abundance of *Vibrio* genus and phosphate concentration are derived from the Taraocean database).

**REFERENCE**

1.Grasshoff K, Ehrhardt M, and Kremling K. 1983. Methods of Seawater Analysis. 2nd Edn. Weinheim: Verlag Chemie.

2. Dai S, Guo J, Liu WW, Liu JX, Ding X, Quan QM, Tan YH. 2024. Labyrinthulomycetes thrives in organic matter-rich waters with ecological partitioning in the Pearl River Estuary. Appl Environ Microbiol 90:e0207523.

3.Mo Y, Peng F, Gao X, Xiao P, Logares R, Jeppesen E, Ren K, Xue Y, Yang J. 2021. Low shifts in salinity determined assembly processes and network stability of microeukaryotic plankton communities in a subtropical urban reservoir. Microbiome 9:128.
